# The Essence of Authenticity

**DOI:** 10.3389/fpsyg.2020.629654

**Published:** 2021-01-21

**Authors:** Olaf Dammann, Katja M. Friederichs, Sabine Lebedinski, Kerstin M. Liesenfeld

**Affiliations:** ^1^Liesenfeld Research Institute, Boston, MA, United States; ^2^Department of Public Health and Community Medicine, Tufts University School of Medicine, Boston, MA, United States; ^3^Department of Gynecology and Obstetrics, Hannover Medical School, Hannover, Germany; ^4^Department of Differential Psychology, University of Trier, Trier, Germany; ^5^Institute of Psychology, University of Osnabrück, Osnabrück, Germany

**Keywords:** authenticity, congruence, self, self-development, continuity in self-development

## Abstract

In this paper, we build upon the model of authenticity proposed by Lehman and colleagues, which includes the dimensions consistency, conformity, and connection. We expand this “3C-view” by adding a fourth dimension, continuity, which results in what we have come to call “4C-view of authenticity.” We discuss our proposal from a process perspective and emphasize that congruence might be a reasonable candidate for a concept that unifies the four dimensions of authenticity.

## Introduction

In a recent review article, Lehman and colleagues write about authenticity in the context of management studies (Lehman et al., 2019). Their point of departure is their perception that the term “authenticity” refers to what is real, genuine, or true. In contrast to this, Lehman and coauthors outline their perspective of authenticity as dependent on the *referent* of the term. In keeping with other recent publications motivated by a “lack of definitional clarity” (Newman, 2019, p. 8), they outline different meanings the term can have in different contexts and ask what we are talking about when we talk about something or someone authentic.

Obviously, this semantic heterogeneity has its disadvantages, of which Lehman et al. (2019) emphasize two: the difficulties it brings for scholarly discourse and the possibility of “missing the big picture” (Lehman et al., 2019, p. 2). In order to overcome these hurdles, the authors propose a conceptual framework that rests on three different “meanings” of authenticity: authenticity as consistency, as conformity, and as connection.

In brief, authenticity as *consistency* is the congruous relationship between an entity’s external characteristics and its internal values. Authenticity as *conformity* is a congruous relationship between an entity and its social norms. Authenticity as *connection* is the congruence between an entity and “a person, place, or time as claimed” (Lehman et al., 2019, p. 3). We deliberately use the terms *congruence* and *congruous* in all three definitions because we propose in this paper that congruence is *the essence of authenticity*. In general, we use these terms in keeping with Merriam-Webster’s dictionary definition for *congruous*, “being in agreement, harmony, or correspondence” (Congruous, 2020). We think that all three of Lehman and coworkers’ interpretations of authenticity encompass some form of congruence: a particular congruous relationship between an entity and characteristics of itself, between an entity and its social context, and between an entity and another one, respectively.

We think of authenticity not as a static concept, but as a developmental process, as subject to change. Consequently, we suggest that besides the above three “C’s,” there is a fourth “C,” a fourth meaning of authenticity as *continuity*. The continuity perspective captures the developmental character of authenticity, the ever-changing relationships between an individual and himself/herself, others, and the social norms his/her life is embedded in. These relationships are different across the lifespan; they are different for the same person as a child, adolescent or adult. Hence, authenticity as continuity describes the congruous relationship between an entity and features of development and, therefore, captures the evolving nature of authenticity. Going beyond the static view of authenticity allows for inherent changes in authenticity over time and places a greater emphasis on *becoming* authentic instead of *being* authentic. Thus, authenticity as continuity combines a dynamic connection between static and process characteristics of authenticity, which in turn makes authenticity more of an on-going project instead of something that can be achieved.

Based on these considerations, we define authenticity as “the process of being in a congruous relationship with self, others, and relevant social norms.” Thus defined, we restrict our discussion to authenticity as a concept that can be applied at both the level of the individual as well as at the level of the collective (community and population). In this paper, we first briefly review Lehman et al.’s (2019) view of authenticity as consistency, conformity, and connection (henceforth, “3C-view” of authenticity). Then, we propose the addition of a fourth C, continuity (4C-view). Next, we explore and propose congruence as the essence of authenticity. Finally, we discuss advantages and disadvantages of our proposal.

## The 3C-View of Authenticity

Lehman et al. (2019) tackle the problem of multiple interpretability of the term “authenticity.” The paper appeared in *Academy of Management Annals* and is, therefore, written from an economics and management perspective. In this section, we outline their discussion and argument step-by-step.

The authors’ 3C-View emerges from three guiding questions that address the referent, the target, and the audience of authenticity. First, they argue that in order to capture the meaning of authenticity one has to know who or what is the reference point to assess authenticity, which can be either inside (i.e., the entity itself) or outside of the entity (e.g., social category) that is judged. Second, the meaning of authenticity also depends on who or what the judgment of authenticity is directed to, meaning who or what the entity in itself is (e.g., individuals, organizations, brands, and objects etc.). Lastly, the audience that makes the authenticity judgment should be considered, and whether the audience is congruent with the entity (e.g., the self) or different (e.g., consumers judging authenticity of a product). The concept of authenticity as consistency was derived from classical philosophical works by the ancient Greeks and the existentialist movement (see Lehman et al., 2019). Here, the referent and target are the same (i.e., the entity) and hence authenticity is judged by the congruous relationship between external characteristic and internal representations of values and beliefs of the entity. An entity is primarily considered an individual in this conceptualization. The audience that judges on the congruence can be congruent with the entity (i.e., the alignment of an individual’s internal representations with his/her behavior leads to a feeling of authenticity or inauthenticity) or different (i.e., the perception of someone’s behavior being aligned with his/her assumed inner representations). However, in both cases the interpretative nature of authenticity is very subjective, as internal representations cannot be measured objectively, but are rather dependent on subjective feelings, or estimates from the perceived characteristics. Definitions in this conceptualization of authenticity reflect these two major aspects for alignment: the understanding of one’s true self (i.e., internal representation) and behaving and interacting accordingly (i.e., external characteristics; e.g., Goldman and Kernis, 2002; Harter, 2002; Kernis and Goldman, 2005, 2006; Wood et al., 2008). This conceptualization is informed by research themes around self-concept (Kraus et al., 2011), self-representation (Walumbwa et al., 2008; Hochschild, 2012) and organizational as well as brand identity (Beverland, 2005; Hatch and Schultz, 2017; Lehman et al., 2019).

Understanding the meaning of authenticity as conformity is embedded in cognitive psychology on schemas and work in sociology on institutional categories (for a brief overview see Lehman et al., 2019). Here, the referent lies outside of the entity (i.e., the social category). The target of the authenticity judgment, i.e., the entity, is either an individual (e.g., a musician creates music that is congruous with their genre; a leader with a specific leadership style and their actions are congruous with expectations of that category) or an object (e.g., a restaurant that serves cuisine that is congruous with the restaurant’s theme). Thus, authenticity is assessed by the congruous relationship between the entity and the social category that serves as a referent. The audience that judges on the congruence can be the entity itself or outside of the entity using categories and classifications to locate and evaluate authenticity. They can even act jointly to determine how authentic an entity is (e.g., people rate authenticity of a restaurant by evaluating who dines at that restaurant). As referent and target can both be captured objectively, the interpretative nature is much more objective than in the previous evaluation of authenticity. Yet, according to the authors, it still has subjective elements, as humans define and perceive social categories, and therefore there are some variabilities depending on the audience. In this conceptualization, the referent is dynamic as it is defined by members of the specific category, as they determine the norms and expectations the entity is subject to. Therefore, authenticity is subject to change according to the evolvement of a social category. Authenticity thus conceived “… reflects a concern with correct classification” (Davies, 2001, p. 203; citation in Lehman et al., 2019, p. 14). This conceptualization of authenticity is informed by research themes around category membership (Kovács et al., 2014) and reinterpretation (Negro et al., 2011; Lehman et al., 2019).

The last conceptualization according to the 3C-view is authenticity as a connection, which is derived from work both in psychological essentialism and semiotics (Lehman et al., 2019). Here, the referent is outside of the entity and is identified as a point of connection to a specific origin (i.e., person, place, or time). The target of the authenticity is mainly considered an object (e.g., artwork, clothing, and jewelry). Authenticity is judged by the congruence of the entity and the spatial and/or temporal distance to a specific outside criterion. The audience that judges the authenticity is equally outside of the entity and either relies on expert knowledge (e.g., an authentic Picasso painting is defined by certain criteria) or is the expert in itself (e.g., a connoisseur), which in turn makes the interpretation of authenticity highly objective. This conceptualization is informed by research themes around provenance (Dutton, 2003), transference (Grayson and Martinec, 2004) and symbolism (Leigh, 2006; see also Lehman et al., 2019 for an in depth review).

## The 4C-View of Authenticity

While the 3C-view reflects the complexity of authenticity, it does not capture the developmental aspect of authenticity. Thus, we propose a fourth “C”: authenticity as continuity. This view takes a *process perspective*: it captures the developmental process of authenticity. This perspective goes hand in hand with the remarks on authenticity of Peterson (2005) who characterized the term “authenticity work,” stating that individuals continuously work on appearing and remaining authentic and thus derived that authenticity “[…] is subject to continual change” (Peterson, 2005, p. 1086). Moreover, Koole and Kuhl (2003) hold that a chronic fixation (i.e., being static in authenticity) is rather suboptimal, as a dynamic change in affect and temporary alienation are relevant to allow for self-development and “optimal functioning sometimes requires active suppression of the authentic self […]” (Koole and Kuhl, 2003, p. 46). We think that the study of the variability of authenticity expressions over time, both intra-and inter-individually, will be as relevant to personality psychology research as is the variability of other behavioral characteristics. In particular, conceiving of expressions of authenticity as density distributions over time (Fleeson, 2001) might be one fruitful way of looking at the continuity dimension of authenticity.

Several considerations are important when thinking of authenticity as continuity. First, authenticity is established through repeated self-assessment. An individual has to continuously evaluate whether he/she considers himself/herself being authentic or not. This involves constantly seeking “one’s truth of […] feelings and desires” (Krause, 2017, p. 5f) and reflecting critically on them in order to evolve authentically. Second, in a process, change abounds. Therefore, there are no characteristics of authenticity that are constantly fixed. Everything moves and is always subject to change. This requires the ongoing evaluation and re-evaluation of these ever-changing characteristics with the question in mind whether they fit the then current perception of authenticity. This resonates with the reflections of education researcher Pauline E. Leonard, who wrote about navigating the road of authenticity that “[b]ecoming authentic is a process, a journey, not an end in itself; it is an inner and outer journey and requires a *continual* examination of one’s multiple identities within the context of the communities in which one lives, works, and interacts” (Leonard, 2005, p. 7f). Third, with each change, the question comes up whether the continuity of authenticity is interrupted. It is difficult, but not impossible, to define breakage points where someone considers himself/herself authentic before and non-authentic after such point if the characteristics of authenticity are constantly changing. For example, traumatic events may cause an interruption in authenticity. Quade et al. (2019) examined in a qualitative study about scapegoating – a term that describes actively deflecting the blame received for one’s own failures by blaming others – how this traumatic event left it challenging for female leaders to remain authentic. In their qualitative study, all participants experienced an incongruence between their self-image and the image their audience had of them (Quade et al., 2019). Fourth, every process has a beginning and an end. It seems clear that the authenticity process does not begin at birth due to the intellectual requirements of the person to self-evaluate. The endpoint of the process is hard to determine and is probably only individually defined, perhaps just like all of the process. One could argue that certain illnesses, such as dementia, can lead to an assessment of an endpoint, whereas authenticity is no longer experienced or perceived. Yet as the mental state of people with dementia is fluctuating, it could be considered more as a fading of authenticity rather than an actual end point of authenticity (see also Holm, 2001).

The concept of authenticity as continuity can be viewed in light of the work of modern philosophers, such as Parfit (1984), who addressed moral, personal identity, and normative ethics. In keeping with our outline of the 3 C’s, we apply to the 4th C (authenticity as continuity) the same three guiding questions, considering the referent, the target, and the audience of authenticity. Here, the referent is considered the feature of development over time (e.g., typical development). The target of the identity judgment, i.e., the entity, is mostly considered an individual but can also be an object (e.g., a company), a community, or even a population. Therefore, authenticity is assessed by the congruous relationship between the entity and the feature of development over time that serves as a referent. The audience that judges on the congruence can be the entity itself or reside outside of the entity, who either rely on expert knowledge on features of development or the “gut feeling” of how oneself or others stay true to their path of development. While the former can be considered rather objective, the latter is very subjective since internal representations cannot be measured objectively, but are rather dependent on subjective feelings, or on inferences based on perceived characteristics. This conceptualization is informed by research themes that revolve around personality development, subjective sense of self, and individual values (Kuhl, 2001, 2020; Newman, 2019; Urminsky and Bartels, 2019).

Taken together, our extension of Lehman’s 3C-view of authenticity provides a comprehensive model of authenticity, henceforth called *4C-Model* of authenticity (Table 1). The 4C-model allows for a more complete, inclusive, and integrated understanding of authenticity. Moreover, the model reveals interesting relations among the different characteristics of authenticity.

**Table 1:** The 3-Cs proposed by Lehman et al. (2019) and the fourth C proposed in this paper.

C #	Dimension	Relationship between an entity’s/individual’s self and the referent below	Individual level example
1	Consistency	external characteristics of self (e.g., behavior)	A person who is true to themselves
2	Conformity	social norms	An individual who lives in harmony with, and according to the rules accepted by their community peers
3	Connection	socio-spatial-temporal position	A person who is well-integrated in their community and live their life fully embedded in its history, value system, and expectations
4	Continuity	features of development	An individual whose changes over their lifespan are in keeping with what is socially accepted as “typical development”

Dimensions are explained by referent and by example.

**Figure 1:**
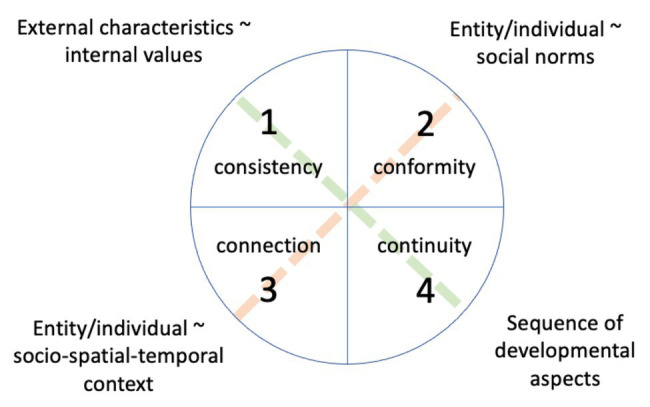
The 4-C model of authenticity with personal axis (green diagonal) and social axis (red diagonal) displayed in juxtaposition. The personal axis refers to the congruence of internal values and external characteristics (C1) and the congruence of the individual’s personal development with what is expected to be typical in their community (C4). The social axis refers to the congruence of the individual and the social norms of their community (C2) and the congruence of the individual and their social position (C3).

All four Cs are relational in that they either relate different kinds of characteristics of one individual or entity to each other (C1, C4) or characteristics of an individual or entity to something external (C2, C3; Figure 1). All four Cs are arranged so that these two axes of authenticity relations self-self (green) and self-world (red) form the diagonals (1,4) and (2,3), respectively. What all four kinds of relationship have in common is that the relation is congruous.

## Congruence as the Essence of Authenticity

In this section we explicate what we mean when we use the term *congruence* in our present context. We also outline why we think that congruence is the *essence of authenticity*.

The Latin origin of the word, *congruere*, means *to come together*, *to fit in*, *to correspond*, or *to agree*. We see things or aspects as congruous or congruent when we *see fit*. When characteristics that form or represent the relata of C–relationships *go well together*, we consider them congruous. What does that mean, to go well together? Let us consider a few examples pertinent to C1, consistency.

First, it should be possible to assess (evaluate) the relata and their relationship. We cannot establish congruence if we cannot make up our mind about the meaning of relata in and of themselves, as well as for each other. In terms of consistency (C1), what are we talking about when we talk about external features and internal values? We need to define what counts as external and internal value. We also need to explicate what external feature and internal values we are talking about.

Second, the relata have to be mutually relevant. For example, the relationship between external characteristics and internal values in C1 should have mutually relevant relata if, for example, a person behaves in a friendly and peaceful manner and his/her values include a devotion to non-violence. It makes less sense to attempt to establish a relation between peaceful behavior and the part of a person’s value system that embraces family values. Congruence can be evaluated in the former, but not the latter case. We can say that friendly behavior and a non-violent stance are congruent, and that aggressive behavior and a non-violent stance are incongruent. But we cannot say that friendliness and family values are necessarily congruent. On the other hand, aggressive behavior and family values are not entirely incongruent.

Third, and most obviously, the relata have to support each other in a justificatory, explanatory, or even causal way, at least in one, sometimes in both directions. A non-violent stance causes peaceful behavior, which in turn supports a non-violent stance. Anti-racist values explain inclusive behaviors, which in turn nurture an anti-racist value system.

Fourth, the relata should not contradict each other. Supporting the death penalty and insisting on keeping the 10 Commandments as an important component of one’s value system is a contradiction. Only if relata stand in a non-contradictory relationship with each other can we consider them congruous.

In sum, we see congruence as referring to a self-self or self-world relationship in which relata are assessable, mutually relevant and supportive, and non-contradictory. Based on our definition of congruence and our discussion of the 4 C’s we believe that congruence is a formidable candidate for the common denominator of all four Cs and, therefore, for representing the essence of authenticity.

## Discussion

In this paper, we have discussed Lehman’s 3C view of authenticity and expanded it by adding a fourth C, continuity. This modification emphasizes our view that authenticity is not static, but a process. This *process perspective* is based on the assumption that authenticity is subject to change, requires continuous work, and can thus be characterized as a developmental process.

Moreover, we have proposed that congruence may be the essence of authenticity. All four Cs require congruent relationships between internal and external aspects and, together, represent a proposed 4C model of authenticity.

Rogers uses the terms “congruence” and “incongruence” to delineate the difference between individuals who live an authentic life (congruence) at least in part due to receiving positive regard and those who cannot (incongruence) and who develop defense mechanisms (Rogers, 1951). According to Rogers’ concept of an “organismic valuing process,” a person has an inborn capability to estimate what kinds of changes will be good for them in terms of being conducive to their strive towards such lived congruence. Sheldon and colleagues have put that theory to the test and performed a study to see “how people change their minds over time about what goals and values to pursue” (Sheldon et al., 2003, p. 837). Their results suggest that individuals shift towards intrinsic more than extrinsic goals, which supports the hypothesis that subjective well-being plays a greater role in such changes than, e.g., social desirability. These results provide an illustration of the developmental process we aim to capture with our fourth C, continuity. They also suggest that Lehman et al.’s consistency might be a stronger motivation for such developmental change than conformity.

Among the many possible repercussions of *inauthenticity* appears to be that experiencing inauthenticity can come as a threat to one’s moral self-concept (Gino et al., 2015). Gino and colleagues have offered the explanation that inauthenticity and dishonesty share similar roots in that both are a “violation of being true, whether to others or oneself” (p. 984). Feelings of impurity in the context of inauthenticity could be explained by a spillover effect, because dishonesty is not socially accepted, while inauthenticity is. However, experiencing inauthenticity (i.e., incongruence) is a vital aspect for the process perspective of authenticity, as incongruence allows for continuous re-evaluation, and hence offers opportunities for development (Kuhl, 2020).

How does our 4C-model of authenticity fit with existing categorization schemes? Newman recently lamented the “lack of definitional clarity [which is] due in part to the diversity of contexts in which authenticity is studied” (Newman, 2019, p. 8). In response to this perceived heterogeneity of definitions, Newman proposes three broad categories, i.e., historical, categorical, and values authenticity. The first two apply mainly to objects (e.g., pieces of art or types of food, resp.) Only the third appears to be applicable to individuals in that it refers to “the consistency between an entity’s internal states and its external expressions” (Newman, 2019, p. 10). This definition maps directly onto Lehman et al.’s first dimension of authenticity. Both Lehman et al.’s three and our fourth dimension should not be viewed as mutually exclusive categories, but as viewpoints or lenses through which the different kinds of authenticity can be studied.

We wish to emphasize that we developed our 4C-model of authenticity in the context of models of the self. In other words, we refer to authenticity (in the present context) as a characteristic of the self and its development. Our goal is not to contribute to the debate about what authenticity is *in general*, but to expand the list of characteristics of authenticity in the particular context of concepts of the self and the development of the self. When Newman writes about the “psychology of authenticity,” his focus is on what he calls “lenses” or “dimensions of consideration” (Newman, 2019, p. 10). His lenses (historical, categorical, and value) are not characteristics or dimensions of authenticity, but of the ways how authenticity is established in different contexts.

Carroll views authenticity as “an attribution – nothing more, nothing less” (Carroll, 2015, p. 3). He claims that “[i]n modern society, authenticity is often socially constructed,” and is thereby “culturally contingent and historically situated” (Carroll, 2015, p. 3) He contrasts this kind of socially constructed authenticity with Dutton’s *nominal authenticity*, e.g., the kind of authenticity attributed to an original painting or historically authentic piece of clothing (Dutton, 2003), the authenticity of which can usually be “objectively and definitively evaluated”(Carroll, 2015, p. 4). It remains to be explored whether the individual authenticity (the authentic self) we discuss in this paper is a kind of nominal authenticity that can be evaluated, but only subjectively, or if it is culturally contingent, or both.

Given that we have developed our model in the area of personality psychology, we do not see it as representing a different category, but as an additional dimension that, together with Lehman et al.’s three Cs, applies mainly to Newman’s category of values authenticity. We hope that our model will be helpful in further research on authenticity not only in individuals, but perhaps also in communities and populations. Such extension of our model from the realm of personal to social psychology, however, is clearly beyond the scope of this paper.

## Data Availability Statement

The original contributions presented in the study are included in the article/supplementary material, further inquiries can be directed to the corresponding author.

## Author Contributions

OD is the primary contributor regarding the proposed 4-C model and its outline. KL contributed to the overall idea and specific topic of this publication. All authors contributed to the writing of article and approved the submitted version.

### Conflict of Interest

The authors declare that the research was conducted in the absence of any commercial or financial relationships that could be construed as a potential conflict of interest.
